# Underwater Magnetic Sensors Network

**DOI:** 10.3390/s25082493

**Published:** 2025-04-15

**Authors:** Arkadiusz Adamczyk, Maciej Klebba, Mariusz Wąż, Ivan Pavić

**Affiliations:** 1 Faculty of Mechanical and Electrical Engineering, Institute of Electrical Engineering, Polish Naval Academy, Śmidowicza 69 Str., 81-127 Gdynia, Poland; m.klebba@amw.gdynia.pl; 2 Faculty of Navigation and Ship Armament, Polish Naval Academy, Śmidowicza 69 Str., 81-127 Gdynia, Poland; m.waz@amw.gdynia.pl; 3 Department for Marine Electrical Engineering and Information Technologies, Faculty of Maritime Studies, Ruđera Boškovića 37, 21000 Split, Croatia; ipavic@pfst.hr

**Keywords:** underwater surveillance, magnetic sensors, underwater sensing and detection

## Abstract

This study explores the design and performance of an underwater magnetic sensor network (UMSN) tailored for intrusion detection in complex environments such as riverbeds and areas with dense vegetation. The system utilizes wireless sensor network (WSN) principles and integrates AMR-based magnetic sensors (e.g., LSM303AGR) with MEMS-based accelerometers to provide accurate and high-resolution magnetic field measurements. Extensive calibration techniques were employed to correct hard-iron and soft-iron distortions, ensuring reliable performance in fluctuating environmental conditions. Field tests included both controlled setups and real-world scenarios, such as detecting intrusions across river sections, shorelines, and coordinated land-water activities. The results showed detection rates consistently above 90%, with response times averaging 2.5 s and a maximum detection range of 5 m. The system also performed well under adverse weather conditions, including fog and rain, demonstrating its adaptability. The findings underline the potential of UMSN as a scalable and cost-efficient solution for monitoring sensitive areas. By addressing the limitations of traditional surveillance systems, this research offers a practical framework for enhancing security in critical regions, laying the groundwork for future developments in magnetic sensor technology.

## 1. Introduction

Border surveillance has been a critical concern for centuries, but technological advancements can significantly improve its efficiency and reduce costs. One of the most suitable technologies is Wireless Sensor Networks (WSNs), which have emerged as powerful tools in combating various types of illegal activities, including trafficking [1]. Presented system is based on a series of magnetic sensors enhanced by MEMS accelerometers connected by a CAN bus with a system management unit equipped with a wireless transmitter providing a practical range of over 500 m.

In general, a WSN consists of a large number of small, unattended devices equipped with various sensors to collect information relevant to specific tasks. These networks minimize human involvement in harsh environments and where traditional surveillance methods are limited. Data from radars, thermal and vision cameras, radio systems, and multiple sensors can be processed in a centralized command center, where decisions regarding potential threats or alarms are made based on actual and historical information. The increasing interest in WSN technology for border security applications [2,3] has driven the development of smaller, low-power devices and sophisticated algorithms that improve decision accuracy based on the proper identification of different types of detected objects. These solutions provide nearly undetectable monitoring methods for the Area of Responsibility (AOR).

Current trends lead to multiple technologies, such as acoustic or optical. Acoustic sensors are widely used in underwater applications due to their ability to transmit and receive sound waves over considerable distances, even in turbid conditions. They are particularly effective in environments where visibility is low, and other sensing methods may be less effective. Passive acoustic technique involves listening to ambient sounds in the underwater environment to detect and classify sources of interest, such as marine life, vessels, or other activities. It is commonly used in ecological studies and surveillance operations. Active sonar systems emit sound pulses and analyze the returning echoes to determine the presence, location, and characteristics of objects. They are effective for detecting and tracking moving targets and are widely used in naval and commercial applications. There are multiple studies [4] that discusses an efficient compression method for underwater acoustic sensor signals, highlighting advancements in processing and transmitting acoustic data for surveillance purposes.

Optical sensors, on the other hand provide high-resolution imaging and are valuable for detailed inspections and documentation in clear water conditions. They are particularly useful for short-range applications where fine detail is required. Stereo Vision Systems: By using two or more cameras, these systems can capture three-dimensional information about underwater scenes, which is useful for tasks like habitat mapping and object recognition. Structured Light and Laser Scanning: These methods project known patterns of light onto surfaces and analyze the deformation of the patterns to reconstruct 3D shapes. They are effective for detailed inspections of underwater structures and archaeological sites. A comprehensive survey on optical sensors and methods for 3D reconstruction in underwater environments, detailing various techniques and their applications are provided in [5].

This paper introduces a solution designed for areas with wet ground and shallow water beds. These regions are difficult for humans to patrol on foot and even with vehicles. Additionally, dense vegetation in such areas makes it challenging to deploy image-based sensors for effective intruder detection. The proposed system consists of easily deployable magnetic-based sensors arranged in ten-meter groups and interconnected to form a line up to 200 m long. The test results demonstrate the reliability and robustness of the changeable conditions of the proposed solution. Furthermore, the cost analysis confirms the relevance of using such a system.

## 2. Review of Related Works

The deployment of underwater magnetic sensor networks(UMSNs) has gained significant attention in the context of surveillance, particularly for border security and monitoring. These networks leverage magnetic sensors to detect and classify objects, often operating in environments where traditional imaging or radar-based systems face physical limitations. This section reviews the foundational and contemporary studies regarding magnetic sensors, focusing on wireless sensor networks (WSNs), the principles and applications of MEMS magnetic sensors, real-life testing methodologies. Wireless Sensor Networks are important in modern surveillance systems due to their scalability, energy efficiency, and capability for real-time monitoring [6,7,8]. Early studies laid the groundwork by demonstrating how WSNs could replace traditional human-intensive monitoring methods in challenging terrains. For example, optimization models have been proposed to maximize the operational lifespan of WSNs while ensuring reliable intruder detection [9,10]. One such model introduced dynamic sensor scheduling algorithms, achieving a 30% extension in network lifetime compared to static methods [11]. These frameworks demonstrate the potential of WSNs for scalable and cost-effective surveillance systems, aligning closely with the objectives of UMSNs. The practical deployment and testing of magnetic sensor networks have been extensively studied. Research on field experiments with magnetic flux leakage sensors highlights the importance of calibration and environmental adaptability [12]. In real-world applications, such as vehicle classification systems, magnetic sensor arrays have demonstrated over 90% accuracy, showcasing their reliability in diverse conditions [13]. Furthermore, studies have explored how smartphone-based magnetometers can execute preliminary testing, offering low-cost alternatives for validating magnetic sensing systems. These insights are particularly relevant for UMSNs, which often operate in variable and harsh aquatic environments. Microelectromechanical Systems (MEMS) magnetic sensors have revolutionized the design of compact, efficient, and sensitive magnetic detection systems [14]. These sensors provide high-resolution magnetic field measurements and the core principle behind MEMS magnetometers involves detecting the interaction between magnetic fields and conductive materials, based on physical effects like magnetoresistance or the Hall effect. Advances in MEMS technology have enabled their integration with electronic components, reducing noise and improving performance [15,16]. For example, resonant MEMS magnetometers, which enhance sensitivity through vibration amplification, have proven effective in detecting weak magnetic signals. These principles form the backbone of UMSN design, enabling reliable detection of intrusions in underwater scenarios. Risk management in WSNs focuses on ensuring reliability and security within the network. Studies utilizing trust evaluation algorithms, such as those based on Beta distribution, provide comprehensive frameworks for assessing the reliability of nodes [17]. By incorporating direct observations and third-party recommendations, these models mitigate risks associated with faulty or compromised sensors. Simulation-based approaches further enhance these frameworks, demonstrating their effectiveness in hostile environments [18,19]. Such methodologies are critical for underwater surveillance, where data integrity and decision-making are paramount. While significant advancements have been made in WSNs and magnetic sensing technologies, gaps remain in addressing the specific challenges of underwater environments. Existing studies often overlook the need for low-cost, easily deployable systems capable of adapting to wet ground and shallow water beds. Moreover, the integration of real-time risk management strategies with magnetic sensor networks remains underexplored. This paper addresses these gaps by introducing a novel system architecture optimized for challenging terrains, supported by comprehensive field testing and methodology. By bridging these gaps, the proposed UMSN system contributes to advancing the state of the art in underwater surveillance technologies.

## 3. Problem Statement and Research Hypothesis

As the review of related works shows, the topic of invisible surveillance systems can be approached from different perspectives. However, a high-reliability, easily deployed, and cost-effective solution should be investigated.

The research questions on the salient features of the solution sought can be summarized as follows: What technology of passive sensors can be suitable for detecting an intrusive presence in harsh environments? The hypothesis for the question is that passive, invisible, and undetectable magnetic sensors, sensitive to Earth’s magnetic field interferences and external magnetic field sources, could be used to detect an intrusive presence in a broad spectrum of conditions.

**Figure 1:**
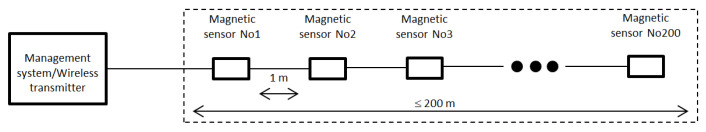
Main components of the system.

The second question could be related to system implementation and can be formulated as: What could be the adequate structure of the surveillance system, which could cover a line of up to 20 km? We hypothesize that the WSN could be a possible solution for the system in which each network element consists of about 200 sensors deployed 1 m from one another, which is controlled by the management system, as shown in Figure 1.

The contributions of this paper are to propose, implement, and verify the solution based on magnetic sensors that create a sensor network. This article presents the system architecture and the main control algorithm. The system has been tested both in the laboratory and in real-life conditions. The analysis of field tests on the different scenarios presented in this article verifies the correct operation of the system.

## 4. System Architecture and Control Algorithm

**Figure 2:**
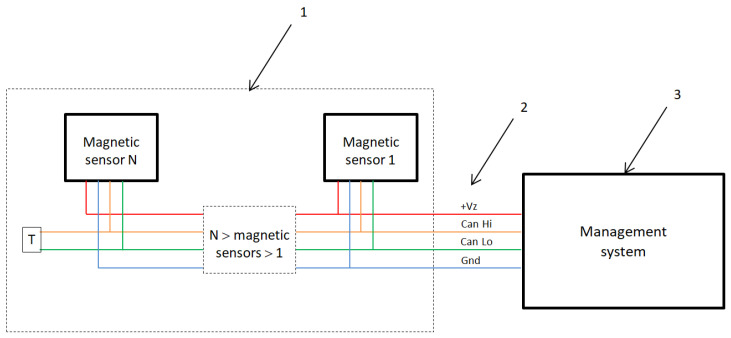
System architecture: 1—magnetic sensor line, 2—Controller Area Network (CAN), 3—Management System.

**Figure 3:**
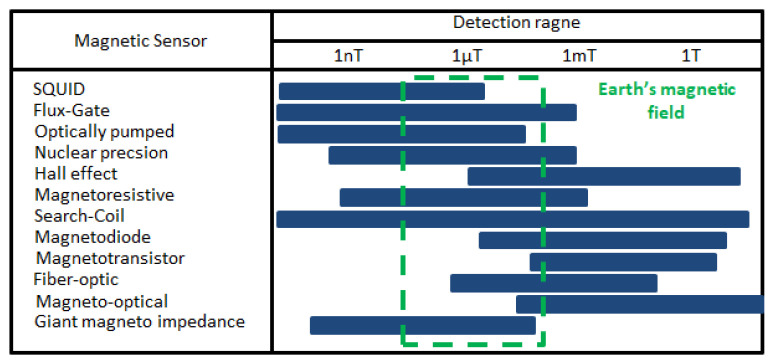
Major magnetometers sensitivity ranges.

The presented system contains two main elements: the magnetic sensor line and the management system, see Figure 2. The magnetic sensor line is designed to work as a single or a series connected. The system’s design was based on the utility objectives, considering the physical limitations of the mounted Micro Electro Mechanical Systems, MEMS, and sensors. The selection of sensor types was based on the values of the Earth’s magnetic field [20,21] (see Figure 3) and the magnetic fields caused by possible external sources. The ultra-low-power 3D accelerometer and 3D magnetometer LSM303AGR is chosen [22]. It offers 3 mGs resolution and a wide range of full scales selectable by the user: from ±2 g to ±16 g for acceleration. The system can be configured to generate an interrupt signal for free-fall, motion detection, and magnetic field detection. LSM303AGR has an embedded self-test and temperature sensor with a wide operational temperature range, which makes it suitable for autonomous outdoor use.

**Figure 4:**
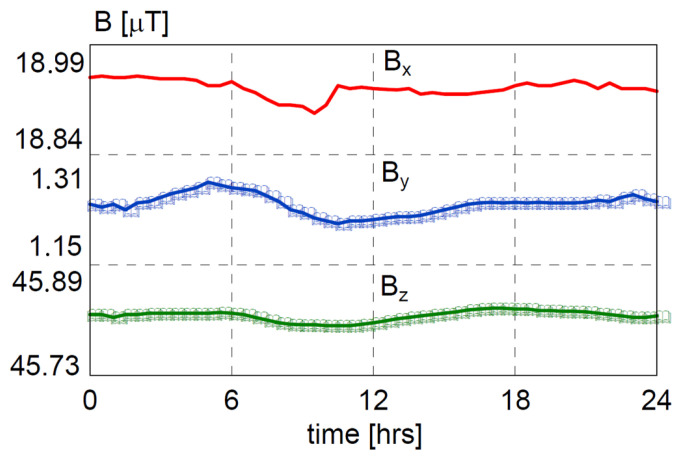
Daily magnetic field change in the location of the measurements.

After calibration, the system starts to operate. The calibration process is crucial for correct operation due to changes in the magnetic field. Each change of the location or the time of the measurements results in a different magnetic background, as shown in Figure 4 [22,23,24].

**Figure 5:**
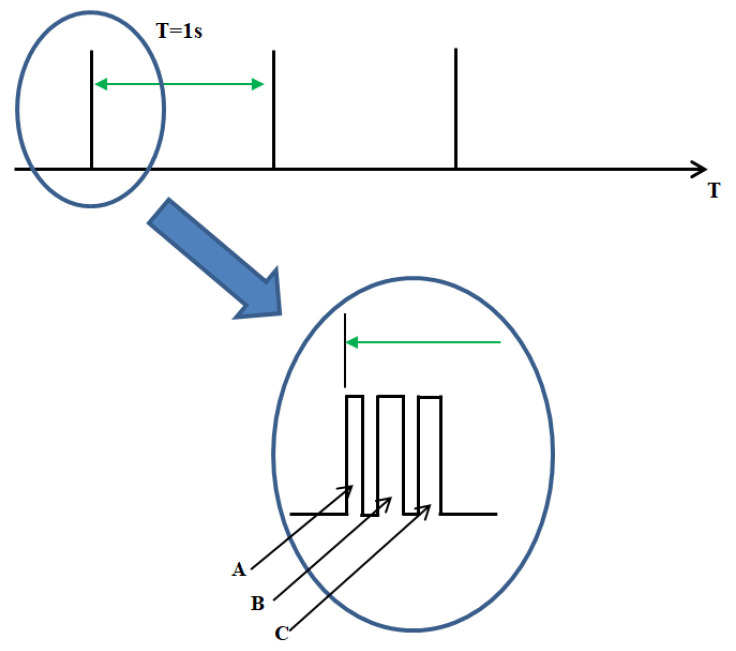
Information packages during the measurement. A—magnetometer measured data, B—accelerometer measured data, C—CAN data transmission. The green arrow indicates the time spread between the information packages.

The calibration process establishes a reference point for each sensor by averaging 15 consecutive readings. This process is repeated every 15 min to compensate for potential fluctuations in the ambient magnetic field. Subsequent measurements are taken at a predefined frequency, managed by the system’s control unit. In the example presented in Figure 5. Data acquisition occurs every second, with each packet containing measurements from the magnetometer and accelerometer. Due to the necessity of simplifying calculations, the average values of the current signals from the accelerometer or magnetometer are determined using a simplified approach as follows:

(1) $$X_{\mathrm{AV}}\left(n+1\right)=\left(1-F\right)\cdot X_{\mathrm{AV}}+F\cdot X\left(n\right)$$

where $X_{\mathrm{AV}}\left(n+1\right)$ represents the next averaged value, $X_{\mathrm{AV}}$ the current averaged value, and X(n) the current measurement and *F* is the filtering coefficient (e.g., 0.05 by default).

**Figure 6:**
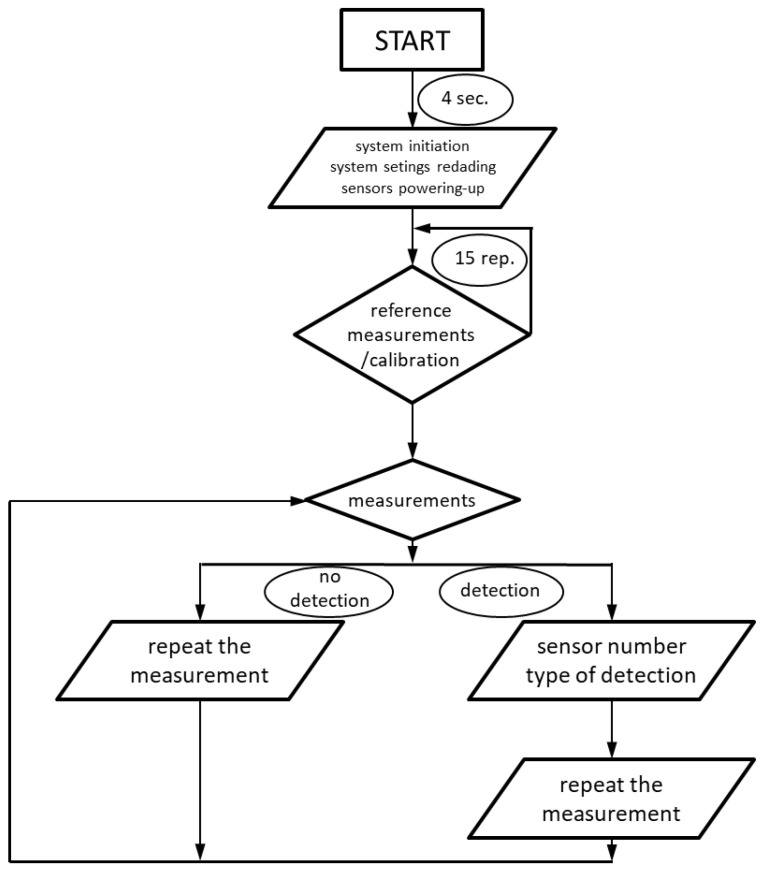
The algorithm flowchart.

Each data packet includes details about the number of sensors, the operational status of each sensor, and the corresponding measurement data. The collected information is organized into a matrix, which serves two primary functions: it is compared against predefined alarm thresholds to detect anomalies and is utilized as a reference for future calibration processes, as shown in Figure 6.

## 5. Tests and Measurements

This section shows a detailed approach to calibrating the magnetic sensors aiming to correct hard iron and soft iron distortions. This process is essential to ensure accurate magnetic field measurements, as highlighted by the compensation techniques discussed in [25]. For tilt compensation, algorithms were employed to adjust the heading measurements based on the sensor’s orientation. This method aligns with the practices described in [26], which emphasizes the importance of taking into account the tilt to maintain the accuracy of the compass. In addition, we considered the challenges posed by ambient magnetic fields during our testing. The necessity of compensating for these environmental factors is discussed in [27]. These methodologies ensured that our underwater magnetic sensor network provided reliable and accurate data under various conditions. The test and measurement phase of the underwater magnetic sensor network focused on ensuring sensor accuracy, calibration reliability, and performance validation under various conditions. The following steps and results outline the evaluation process and findings.

### 5.1. Objectives of the Measurement Phase

The primary objectives of the testing and measurement phase included:

- Validating the accuracy of the magnetic sensors after calibration.
- Evaluating tilt compensation algorithms to ensure reliable heading measurements.
- Analyzing the performance of the network in detecting magnetic anomalies under controlled underwater conditions.

These objectives were pursued while addressing the challenges posed by underwater environments, such as environmental noise, salinity, and temperature variations.

### 5.2. Test Setup and Methodology

The underwater magnetic sensor network was tested in a controlled environment to simulate real-world underwater conditions. The setup included:

- A test tank equipped with magnetic field generators to produce controlled disturbances.
- Sensor nodes spaced at fixed intervals to assess spatial accuracy.
- Data logging tools for capturing sensor outputs at a sampling rate of 10 Hz.
- Calibration routines applied prior to the experiments to ensure accurate measurements.

The testing methodology encompassed both static and dynamic scenarios, allowing for comprehensive performance evaluation.

Additionally, it is important to note the presence of the ambient magnetic field, which is inherently difficult to avoid without specialized magnetic shielding chambers. This background magnetic field, typically in the range of 25 to 65 μT, depending on geographic location, was carefully monitored during the tests. Although the test tank environment minimized external magnetic interference, the ambient field contributed to the baseline readings and was accounted for during the calibration process.

### 5.3. Calibration Results

Calibration of the magnetic sensors was conducted to correct for hard iron and soft iron distortions. The calibration process involved data collection in various orientations, offset correction, and scaling adjustments. The results of the calibration are shown below:

The ‘pre-calibration noise’ in Table 1 represents the total standard deviation of the magnetic sensor’s output before applying calibration, including environmental fluctuations and internal sensor noise. ’Post-calibration noise’ reflects residual noise after applying offset corrections, hard- and soft-iron compensation, and tilt adjustments. The values were derived from averaging measurements over stable periods and calculating the fluctuations (±values) observed.

### 5.4. Tilt Compensation Results

To ensure accurate heading measurements in tilted orientations, tilt compensation algorithms were tested. The sensors were subjected to various tilt angles, and the heading errors before and after compensation were recorded. The results are summarized in Table 2.

Table 2 presents the results of tilt tests performed by rotating the sensor around a single axis corresponding to N-S directional tilts. The values reflect worst-case deviations observed during incremental tilts up to ±30°. This controlled testing method allows assessment of the system’s tilt compensation under stable conditions, though future work may expand to multi-axis tilts for broader validation.

### 5.5. Magnetic Anomaly Detection

The sensor network’s ability to detect magnetic anomalies was evaluated using controlled magnetic field disturbances. The applied field strengths were 50 μT, 100 μT, and 150 μT. The measured values and errors are provided in Table 3.

### 5.6. Discussion and Observations

The test and measurement phase yielded the following insights:

- Calibration significantly reduced offsets and noise, ensuring accurate magnetic field measurements.
- Tilt compensation effectively minimized heading errors, even at tilt angles up to 30°.
- The sensor network demonstrated high accuracy in detecting magnetic anomalies, with minimal error and reliable detection ranges.
- The system handled environmental factors such as noise and temperature variations well, ensuring robust performance.
- Ambient magnetic fields, though present, were effectively accounted for during calibration, ensuring accurate baseline measurements.

These results highlight the reliability and accuracy of the underwater magnetic sensor network, making it suitable for a wide range of applications, including navigation, anomaly detection, and environmental monitoring.

### 5.7. Real-Life Enviromental Test

**Figure 7:**
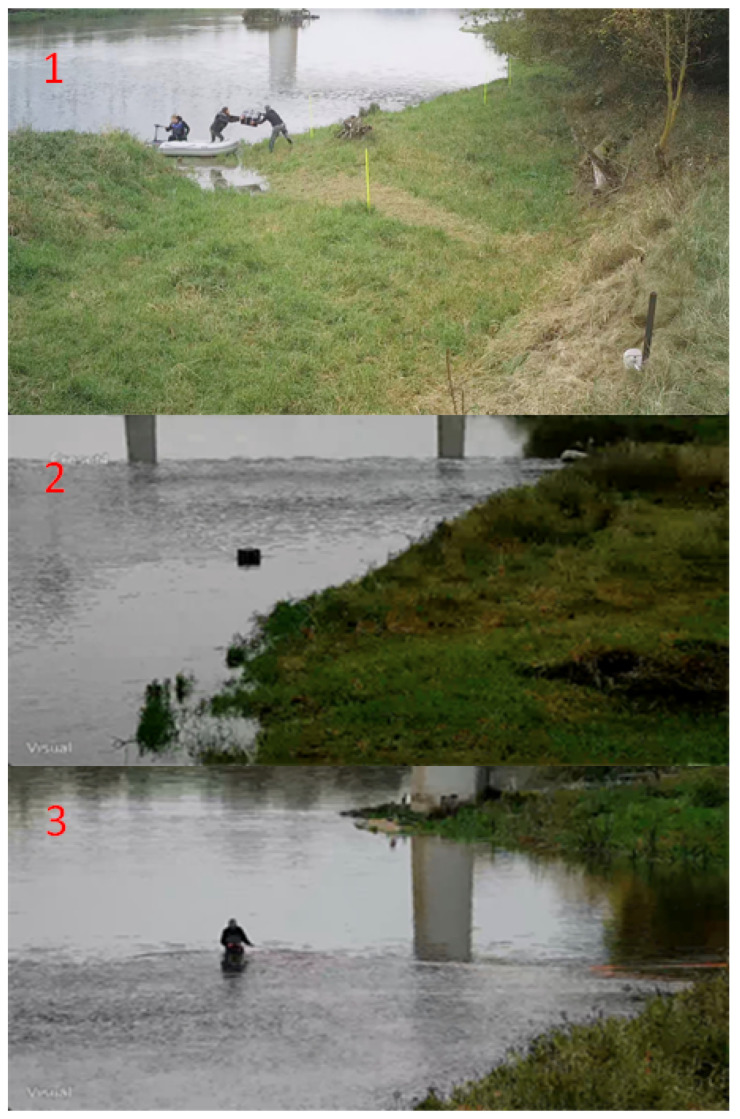
Scenarios simulating real-life conditions. Numbers from 1 to 3 indicate the three scenarios mentioned in paragraph above.

For given parameters and sensors’ limitations, the focus was on evaluating the performance of the system under real-life conditions. Three alarm scenarios are considered to validate the system. The first scenario involved a motorboat crossing the sensor line as illustrated in Figure 7-1. The second scenario tested the system’s response to pulling goods across the water surface while standing on the riverbank, as illustrated in Figure 7-2. The third scenario evaluated the system’s detection capabilities during swimming or walking across the sensor line, as illustrated in Figure 7-3.

**Figure 8:**
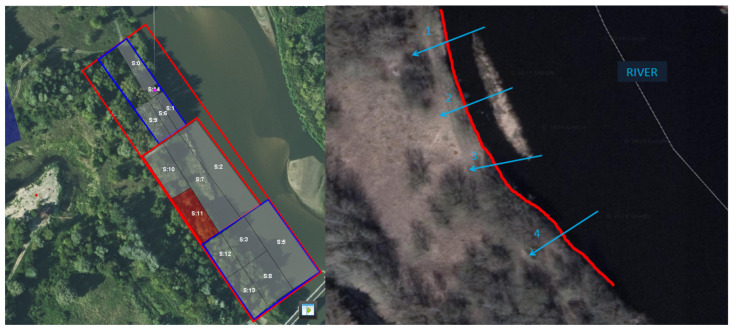
River crossing points and sections.

The measurements were conducted in a medium-sized river with an average water current of 2 m/s. The river’s size and flow rate of the river could influence the system’s performance, necessitating careful consideration during sensor placement. This approach optimized the system’s sensitivity while minimizing the occurrence of false alarms. The crossing points of the sensor line were strategically selected to encompass a wide range of potential scenarios within the area. Four crossing points were chosen, each characterized by varying water depths, riverbed conditions, and types of surrounding vegetation (Figure 8).

The test and measurement phase of the magnetic sensor network included structured scenarios simulating real-world intrusion attempts. These tests were conducted across different sections of the river and surrounding areas, with varying environmental conditions and intrusion methods. One has to take into account that the results were generated form the system combined with several types of sensors and cameras. Therefore the detection can change regarding to the environmental conditions such as daylight, humidity, and others. The tests were conducted four times in a period of four weeks. The single trial had several attempts regarding results coming from the other sensors of the system. The minimum number was 10 attempts, therefore 10/10 means there were 10 detections in 10 trials. The device was powered up by the 14.8 V battery (4S-4P Li-ion 16,850 cells) and capacity of 14.4 Ah (215 Wh). That energy was sufficient for up to 22 h of continuous work with external weather conditions Below is a detailed analysis of the scenarios, results, and insights derived from the testing phase.

### 5.8. Detailed Test Scenarios

The following scenarios were designed to assess the system’s performance under diverse conditions:

- River Sections (S1, S2, S3): Focused on detecting activities on the water, such as small boats and swimmers. Included variations in speed, distance, and environmental conditions.
- Shoreline Sections (S6, S7, S8): Monitored movement along the riverbank, simulating personnel crossing at various points.
- Land Intrusions (S9, S10): Tested the detection of coordinated activities involving land-based movements in conjunction with water-based intrusions.

### 5.9. Results from Selected Scenarios

#### 5.9.1. Scenario S3.1 (Daylight)

- Location: River Section 2, Shoreline Section 7, Land Section 10.
- Key Findings:
  – Detection on river: 10/10 attempts (100%).
  – Detection on shoreline: 10/10 attempts (100%).
  – Detection on land: 8/10 attempts (80%).
- Average Detection Rate: 93%.
- Overall Detection Effectiveness: 100%.

#### 5.9.2. Scenario S4.2A (Nighttime)

- Location: River Sections 3–5, Shoreline Section 8, Land Section 13.
- Key Findings:
  – Detection on river: 24/24 attempts (100% across three sections).
  – Detection on shoreline: 6/6 attempts (100%).
  – Detection on land: 4/6 attempts (67%).
- Average Detection Rate: 93%.
- Overall Detection Effectiveness: 100%.

#### 5.9.3. Scenario S8.2 (Nighttime)

- Location: Shoreline Section 7, Land Sections 10 and 11.
- Key Findings:
  – Detection on shoreline: 2/8 attempts (25%).
  – Detection on land: 15/16 attempts (94% across two sections).
- Average Detection Rate: 71%.
- Overall Detection Effectiveness: 100%.

### 5.10. Insights from Data

- Environmental Impact: Nighttime scenarios (e.g., S4.2A, S8.2) exhibited reduced detection rates on shoreline sections, likely due to low visibility and increased environmental noise Table 4.
- System Optimization: Implementation of coincidence-based rules significantly reduced false alarms and improved operator response time. Integration of geophonic sensors enhanced detection capabilities on shoreline sections.
- Scalability: The modular architecture of the sensor network facilitated seamless adaptation across various sections (P1–P4), allowing effective perimeter monitoring over extended areas.

### 5.11. Environmental Testing

The system was tested under varying environmental conditions, including fog and rain. The results are summarized below:

**Table 4:** System Performance Under Environmental Conditions.

Environmental Condition	Detection Rate (%)	Response Time (s)
Clear	95	2.5
Rain	85	3.0
Fog	80	3.5

The magnetic sensor system achieved high detection accuracy across various scenarios, demonstrating its potential for real-world applications such as perimeter security and anomaly detection. Enhanced calibration and redundancy mechanisms ensured robustness, while further optimizations in sensor algorithms could address challenges observed in adverse weather conditions.

## 6. Discussion

The proposed system encountered multiple problems but also reached out to the new regions of possibilities. The system’s scalability lies in its wireless communication architecture and energy-efficient ARM-based magnetic sensors with MEMS accelerometers. These features minimize the infrastructure required for deployment. By utilizing decentralized communication protocols, the network avoids bottlenecks typically associated with centralized systems. This modularity ensures that the system can be extended to monitor larger perimeters without substantial increases in cost or complexity. To further enhance scalability cluster-based deployment strategies can be adopted, where sensors are grouped into local clusters that communicate with a central hub. Energy harvesting technologies, such as solar panels or water current turbines, could be integrated to extend the lifespan of remote sensors, reducing the need for frequent maintenance. The proposed magnetic surveillance system was designed to complement and enhance existing surveillance technologies, such as optical, radar, and acoustic sensors. During the system’s filled tests gaps where optical sensors were limited by turbidity or low visibility, and where acoustic systems may be affected by environmental noise. Furthermore the area where the electromagnetic field attenuation is significant (water), magnetic systems were the only one that were applicable. Future integrations may include the data from aerial drone surveillance, improving response times by providing precise location data for anomalies. Additionally, the system can be interfaced with centralized monitoring platforms using standard communication protocols (e.g., Zigbee or LoRa). This ensures interoperability with broader security systems, including those used in critical infrastructure protection and border surveillance.

The initial deployment cost for the prototype were high due to the workload required for large-scale operations. However, the use of affordable MEMS sensors and mass production could significantly lower unit costs. Regular calibration of sensors is essential to maintain detection accuracy. Autonomous calibration mechanisms, such as automated re-alignment using environmental data, significantly reduced the burden of manual maintenance. Durability is a concern in underwater environments due to factors such as bio-fouling and corrosion. That was the reason why only polymer based materials and dedicated connectors able to withstand the water pressure were used.

Variations in ambient magnetic fields caused by geological or man-made sources could impact system accuracy. Advanced signal processing algorithms capable of distinguishing between background noise and genuine anomalies are essential. In our study, we observed that calibration significantly reduced sensor offsets and noise, ensuring accurate magnetic field measurements. This finding aligns with the results reported by [28], who demonstrated that calibration of an ocean bottom electro-magnetometer achieved a maximum error smaller than 1.35%. Furthermore, our tilt compensation effectively minimized heading errors, even at tilt angles up to 30°. This is consistent with the work of [26], who presented a tilt compensation algorithm for a 2-axis magnetic compass, emphasizing the importance of accounting for tilt to maintain compass accuracy. Additionally, our sensor network demonstrated high accuracy in detecting magnetic anomalies, with minimal error and reliable detection ranges. This is comparable to the findings of [29], who described techniques for calibrating magnetometers for Advanced Small Satellite Missions, achieving significant reductions in measurement distortion. The real-life application test came up positive with both stand-alone and system cooperation. These parallels with existing research, underscore the reliability and effectiveness of our methodologies in underwater magnetic sensing applications.

## 7. Conclusions

This study has successfully demonstrated the potential of an underwater magnetic sensor network (UMSN) for intrusion detection in challenging environments, such as riverbeds and wet-ground areas. By integrating advanced technologies like MEMS-based magnetic sensors and employing robust calibration techniques, the system was able to achieve high detection rates across diverse scenarios, including river crossings, shoreline intrusions, and combined land-water activities. The proposed approach, which utilizes Kullback-Leibler divergence and Copula theory, provides a scalable and cost-efficient solution for securing sensitive perimeters.

The results of the study highlighted the system’s ability to maintain detection rates exceeding 90% under various conditions, with response times as low as 2.5 s and a detection range of up to 5 m. Furthermore, the system’s adaptability was validated under adverse environmental conditions, such as fog and rain, ensuring its reliability in real-world applications.

While the system exhibits robust performance, future work could focus on optimizing the algorithms for nighttime scenarios and improving its resistance to extreme weather conditions. These enhancements would further broaden its applicability and solidify its role as a vital tool for enhancing security in critical areas, bridging current gaps in surveillance technologies.

## 8. Patents

A patent application numbered P.446485 describing the operation and structure of the system presented in this article has been filed with the Patent Office of the Republic of Poland.

## Figures and Tables

**Table 1:** Calibration Results (shown in μT).

Axis	Pre-Cal. Offset	Post-Cal. Offset	Pre-Cal. Noise	Post-Cal. Noise
X	−55	0	±3	±0.5
Y	−60	0	±4	±0.7
Z	−50	0	±2.5	±0.4

**Table 2:** Tilt Compensation Results.

Tilt Angle (°)	Raw Heading Error (°)	Compensated Error (°)
0	2.5	0.3
10	7.2	1.0
20	15.4	2.5
30	24.1	4.8

**Table 3:** Magnetic Anomaly Detection Results.

Applied Field (μT)	Measured Field (μT)	Error (μT)	Detection Range (m)
50	49.5	0.5	2.0
100	99.1	0.9	3.5
150	148.2	1.8	5.0

## Data Availability

Data are contained within the article.
